# Relapsed nodular lymphocyte-predominant Hodgkin lymphoma presenting as severe paraneoplastic hepatitis: a case report

**DOI:** 10.1186/s13256-023-04014-9

**Published:** 2023-06-30

**Authors:** Anthony J. Deacon, Naeman N. Goetz, Nicholas Weber, Andrew Clouston, Enoka Gonsalkorala, Catherine Baskerville, Barbara Leggett

**Affiliations:** 1 Department of Gastroenterology and Hepatology, Royal Brisbane and Women’s Hospital, Brisbane, Australia; 2 Faculty of Medicine, University of Queensland, Brisbane, Australia; 3 Department of Haematology, Royal Brisbane and Women’s Hospital, Brisbane, QLD Australia; 4 Pathology Queensland, Royal Brisbane and Women’s Hospital, Brisbane, QLD Australia; 5 Department of Internal Medicine and Aged Care, Royal Brisbane and Women’s Hospital, Brisbane, QLD Australia

**Keywords:** Case report, Hodgkin lymphoma, Nodular lymphocyte-predominant Hodgkin lymphoma, Hepatitis, Paraneoplastic hepatitis

## Abstract

**Background:**

Hematological malignancies are an infrequent but important cause of liver dysfunction. There are several mechanisms by which this can occur, including direct malignant infiltration of the hepatic parenchyma and/or vasculature, vanishing bile duct syndrome, and paraneoplastic hepatitis. Paraneoplastic hepatitis is an extremely rare mechanism by which a hematological malignancy can cause liver dysfunction, and we present the first case, to our knowledge, of paraneoplastic hepatitis caused by nodular lymphocyte-predominant Hodgkin lymphoma in the literature.

**Case presentation:**

A 28-year-old Caucasian male presented with 3 weeks of fatigue, epigastric pain, and jaundice. His medical history was significant for early stage nodular lymphocyte-predominant Hodgkin lymphoma in the cervical region in remission for 5 years after primary treatment with involved-field radiotherapy. Liver biochemistry was normal at the time of treatment for lymphoma and there was no known liver disease before the current presentation. On physical examination, there was scleral icterus and ecchymoses, but no evidence of hepatic encephalopathy, other stigmata of chronic liver disease, or lymphadenopathy. A computed tomography scan of his neck, chest, abdomen, and pelvis showed heterogeneous enhancement of the liver, multiple enlarged upper abdominal lymph nodes, and an enlarged spleen with multiple rounded lesions. Portal and hepatic veins were patent. Initial workup for viral, autoimmune-, toxin-, and medication-related hepatitis was negative. A transjugular liver biopsy was performed with histology showing a predominantly T-cell mediated hepatitis with very extensive multiacinar hepatic necrosis, but no evidence of lymphoma within the liver. Retroperitoneal lymph node biopsy revealed nodular lymphocyte-predominant Hodgkin lymphoma. The patient’s symptoms, bilirubin, and transaminases improved significantly after treatment with oral prednisolone and a staged introduction of rituximab, cyclophosphamide, doxorubicin, vincristine, and prednisone chemotherapy.

**Conclusions:**

Nodular lymphocyte-predominant Hodgkin lymphoma may cause paraneoplastic hepatitis. Physicians should be aware of the possibility of this life-threatening presentation and the importance of early liver biopsy and treatment before acute liver failure occurs. Interestingly, paraneoplastic hepatitis did not occur when nodular lymphocyte-predominant Hodgkin lymphoma was first diagnosed and confined to the cervical region, but was the presenting feature of the recurrence below the diaphragm.

## Background

The differential diagnosis of marked elevation in serum aminotransferases is broad, with severe cases of liver injury defined as serum aminotransferases ≥ 15 times the upper limit of normal [1]. Progression to acute liver failure (ALF) is defined when a patient with no preexisting liver disease and a severe hepatocellular or cholestatic liver injury develops synthetic dysfunction including both hepatic encephalopathy and a prolonged prothrombin time (international normalized ratio greater than or equal to 1.5) [2]. This may be associated with other markers of synthetic liver dysfunction including hypoglycemia, lactic acidosis, and hypoalbuminemia. In the Australian population, paracetamol poisoning is the most common etiology of this clinical scenario, accounting for 50% of cases, followed by viral hepatitis and nonparacetamol drug- or toxin-induced liver injury [3]. This pattern is mirrored in other western countries, including the UK and the USA; however, worldwide, viral hepatitis predominates followed by drug-induced liver injury including paracetamol [4]. Causes related to malignancy of extra-hepatic origin are rare and not uniquely reported [3].

Neoplasms of nonhepatic origin frequently present with hepatic involvement; however, severe liver injury and ALF is rare [5–7]. The most common mechanism of liver injury is metastatic disease as the liver is the second most common site of metastasis in adults [8]. This usually causes a mild-to-moderate rise in serum aminotransferases or cholestatic enzymes without synthetic dysfunction. Rarely, diffuse metastatic infiltration of the hepatic parenchyma and/or vasculature can occur in solid organ and hematological malignancies, which can result in severe liver injury and progression to ALF due to extensive hepatocellular necrosis resulting from pressure atrophy and interference of the vascular supply [5–7, 9–11]. With respect to hematological malignancies, vanishing bile duct syndrome due to cytokine release and paraneoplastic hepatitis have also been described as causes of severe liver injury, but are exceedingly rare described only in case reports and small case series [5, 10, 12–14]. Secondary causes of liver injury in patients with malignancy are also common, with cases of viral infection and reactivation, large duct biliary obstruction, and thrombosis of the hepatic and portal veins widely described.

Our case focuses on paraneoplastic hepatitis as the presenting syndrome of recurrence of nodular lymphocyte-predominant Hodgkin lymphoma (NLPHL). Paraneoplastic syndromes occur in up to 8% of patients with malignancy; however, cases of paraneoplastic hepatitis are poorly described [15]. Paraneoplastic hepatitis, like many other paraneoplastic syndromes, is thought to occur when malignant cells present antigens similar to one present on a hepatocyte, leading to either cell-mediated or humoral destruction of hepatocytes. This is analogous the proposed pathogenesis of autoimmune hepatitis, with molecular mimicry and loss of self-tolerance leading to cytotoxic T-cell and humoral-mediated hepatocyte destruction [16]. To our knowledge, this is the third case of paraneoplastic hepatitis as a result of Hodgkin lymphoma in the literature, and the first in a case specifically of NLPHL, with previous cases and treatment challenges being described by Gunasekaran *et al*. [13] and Dourakis *et al*. [14].

## Case presentation

A 28-year-old Caucasian male presented with severe liver injury characterized by jaundice, elevated transaminases, coagulopathy, and hypoglycemia. He described a 3 week history of fatigue, jaundice, and intermittent epigastric pain but denied fevers, sweats, weight loss, or myalgia. He drank less than six standard alcoholic drinks per week and had no history of intravenous or recreational drug use, tattoos, recent travel, or sexual contact beside his wife. He denied taking any regular medications except for an over-the-counter multivitamin purchased at a pharmacy. There was no history of toxin ingestion, including mushroom consumption. He had not used any other herbal or nutritional supplements. There was no family history of liver disease.

His past medical history was significant for NLPHL, Ann Arbor stage IA, diagnosed at age 23 years after he presented with fatigue and right cervical lymphadenopathy. He was treated with involved field radiotherapy 30 Gy in 15 fractions. End-of-treatment positron emission tomography (PET) demonstrated partial anatomical response with residual fluorodeoxyglucose (FDG) avidity in the index node above that of liver, consistent with partial metabolic response (Deauville score 4). Follow-up PET 6 months later showed further reduction in size and FDG avidity of the node, and he was considered to be in clinical remission. Liver function tests throughout this time were always normal. His history was also significant for L5 radiculopathy treated with L4/5 microdiscectomy earlier in the year.

At the time of the current presentation, he appeared visibly jaundiced but was alert and oriented with no asterixis. There was no palpable lymphadenopathy. There were ecchymoses, but no evidence of hepatic encephalopathy or stigmata of chronic liver disease. His abdomen was soft with mild tenderness on deep palpation but no appreciable organomegaly or ascites. Examination was otherwise unremarkable, including a normal neurological examination and no evidence of congestive cardiac failure. His body mass index (BMI) was 28.7 kg/m^2^.

Laboratory results on admission were as follows: total bilirubin of 156 μmol/L (normal range < 20 μmol/L), direct bilirubin 100 μmol/L (< 4 μmol/L), alanine transaminase (ALT) 2190 U/L (< 45 U/L), aspartate transaminase (AST) 1750 U/L (< 35 U/L), alkaline phosphatase (ALP) 143 U/L (30–110 U/L), gamma-glutamyl transferase (GGT) 214 U/L (< 55 U/L), albumin 36 g/L (35–50 g/L), international normalized ratio 1.9 (0.9–1.2), ferritin 3380 μg/L (40–440 μg/L), C-reactive protein (CRP) 8.4 mg/L (< 5 mg/L), and globulins 26 g/L (25–45 g/L). His full blood count was unremarkable, with a hemoglobin of 157 g/L, white cell count of 4.4 × 10^9^/L, and platelets of 160 × 10^9^/L. Viral serologies including hepatitis A, B, and C; human immunodeficiency virus, Epstein–Barr virus, cytomegalovirus, and herpes simplex virus were negative. He had a negative antinuclear antibody screen and serology for antismooth muscle, antimitochondrial, and anti-liver/kidney microsomal antibodies were negative. Furthermore, he had normal alpha-1-antitrypsin genotype and normal serum copper and ceruloplasmin levels. Peripheral blood flow cytometry detected no monoclonal B cells.

An ultrasound of his abdomen demonstrated a heterogeneous liver with a span of 14 cm, gallbladder wall thickening without cholelithiasis or biliary obstruction, and splenomegaly with a diameter of 15 cm. The portal vein was patent, and the hepatic veins were not visualized. A computed tomography scan of his neck, chest, abdomen, and pelvis showed a heterogeneous enhancement pattern within the liver, multiple enlarged upper abdominal lymph nodes, and an enlarged spleen with multiple rounded lesions. No discrete liver lesions were observed. Portal and hepatic veins were patent although the left hepatic vein was not well visualized. There was no evidence of intraabdominal lymphadenopathy compressing the hepatic vasculature. There were no significant findings in the chest or in the neck at the site of the previous lymphoma. As the ultrasound and computed tomography scan showed no evidence of biliary tree abnormality, magnetic resonance cholangiopancreatography or endoscopic retrograde cholangiopancreatography were not performed. A transthoracic echocardiogram showed normal systolic and diastolic function, nor any other abnormality that would suggest this was a case of congestive hepatopathy.

At this point, clinical consensus was that his lymphoma had recurred, though the exact mechanisms of the liver injury was unclear. The main differentials considered were lymphomatous infiltration of the hepatic parenchyma, a Budd–Chiari syndrome, or an autoimmune-mediated hepatocyte injury. Ultimately, a decision was made on the second day of his admission to proceed with transjugular liver biopsy and a hepatic venogram as this would be able to assess patency of the hepatic veins and potentially provide a tissue diagnosis. On catheterization, there was no significant difference in pressure gradient between hepatic vein and right atrium to suggest Budd–Chiari or hepatic outflow obstruction. The liver biopsy was performed without complication.

**Fig. 1:**
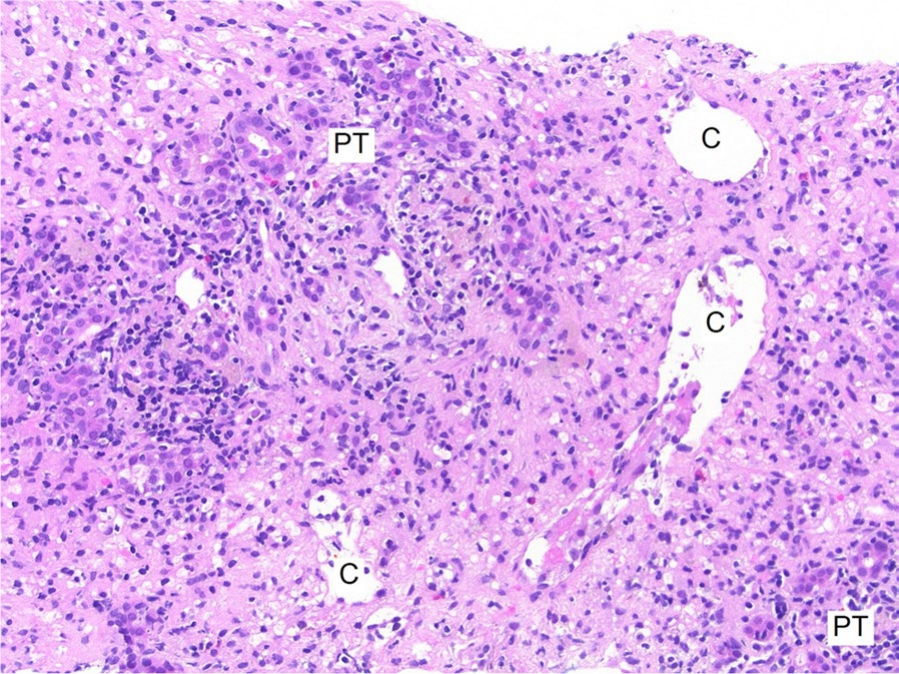
Liver biopsy showing acute hepatitis and multiacinar necrosis. Acute hepatitis and multiacinar necrosis with inflamed portal tracts separated by collapsed reticulin. No hepatocytes remain in the lobules, but central veins (C) are seen in a normal distribution (hematoxylin–eosin, original magnification ×100)

**Fig. 2:**
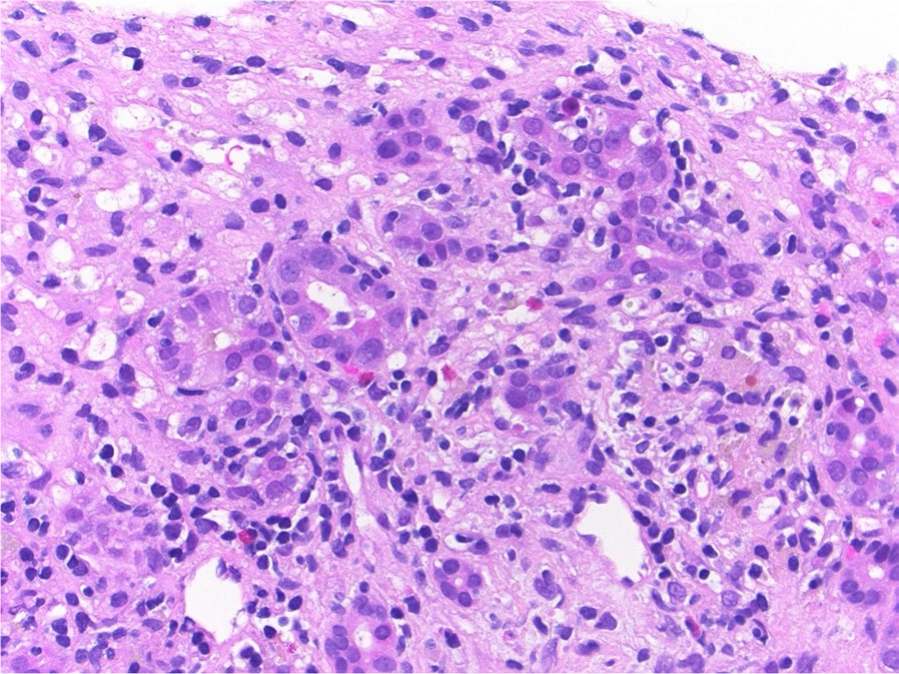
Liver biopsy showing lymphocytic infiltration of the ductular epithelium. There is prominent lymphocytic infiltration of biliary ducts and ductules. The portal inflammatory infiltrate does not contain plasma cells or eosinophils (hematoxylin–eosin, original magnification ×400)

Due to multiple episodes of hypoglycemia (multiple central and peripheral blood glucose measurements < 3 mmol/L) and worsening coagulopathy raising concern for impending ALF, the patient was commenced on prednisolone 1 mg/kg (100 mg) immediately following the liver biopsy. The biopsy showed severe acute hepatitis with multiacinar hepatocyte dropout (Fig. 1). Portal areas showed a regenerative ductular reaction, and there was a moderate lymphocytic inflammatory infiltrate, without evident eosinophils or plasma cells. Lymphocytic infiltration of the ductular epithelium was prominent (Fig. 2) but there was no duct loss. Interface hepatitis was difficult to assess since all hepatocytes in the biopsy cores had been lost, leaving only collapsed reticulin, lymphocytes, and macrophages in the lobular areas, so the limiting plates could not be defined. There was no lymphomatous infiltrate. There was grade 1 iron staining in the few residual hepatocytes with moderate iron staining in hypertrophied Kupffer cells and macrophages consistent with an acute reactive change.

**Fig. 3:**
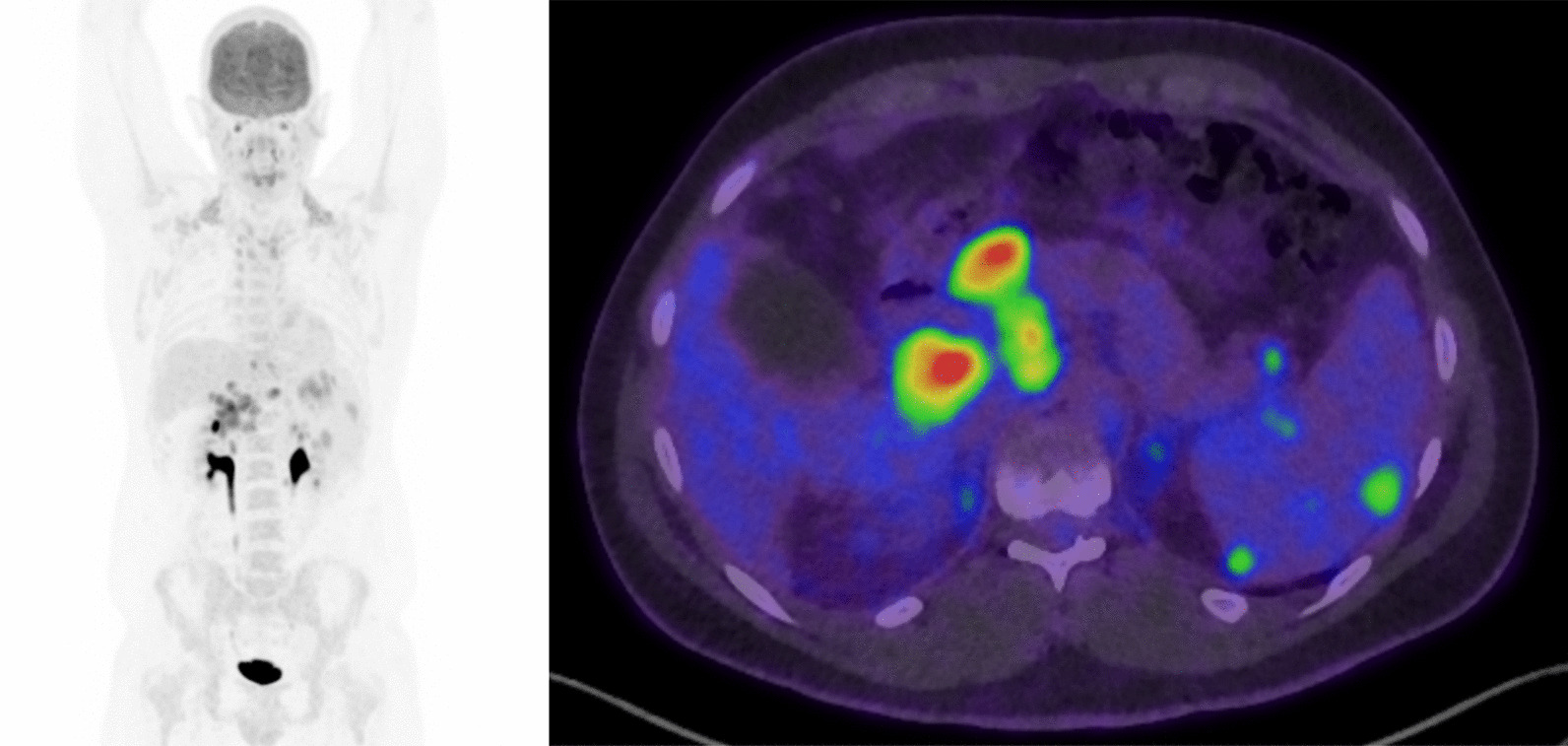
Fluorodeoxyglucose positron emission tomography scan. The fluorodeoxyglucose positron emission tomography scan shows abnormal FDG uptake primarily in the spleen, porta hepatis, and portocaval and paraaortic nodes

PET scan demonstrated above and below diaphragm FDG-avid nodal disease, predominantly within the upper abdomen, as well as lymphomatous involvement of the spleen as evidenced by splenomegaly and multifocal FDG uptake (Fig. 3). There was, however, no evidence of FDG avid lymphomatous hepatic involvement. Imaging-guided core biopsy of a retroperitoneal lymph node revealed sparse CD20+ /PAX5+ large cells surrounded by a polymorphous reactive infiltrate, consistent with recurrent NLPHL of the same phenotype. Interestingly the core biopsy unintentionally included a sample of nearby liver, and there the tissues were able to be clearly distinguished reinforcing the absence of lymphoma within the liver.

**Fig. 4:**
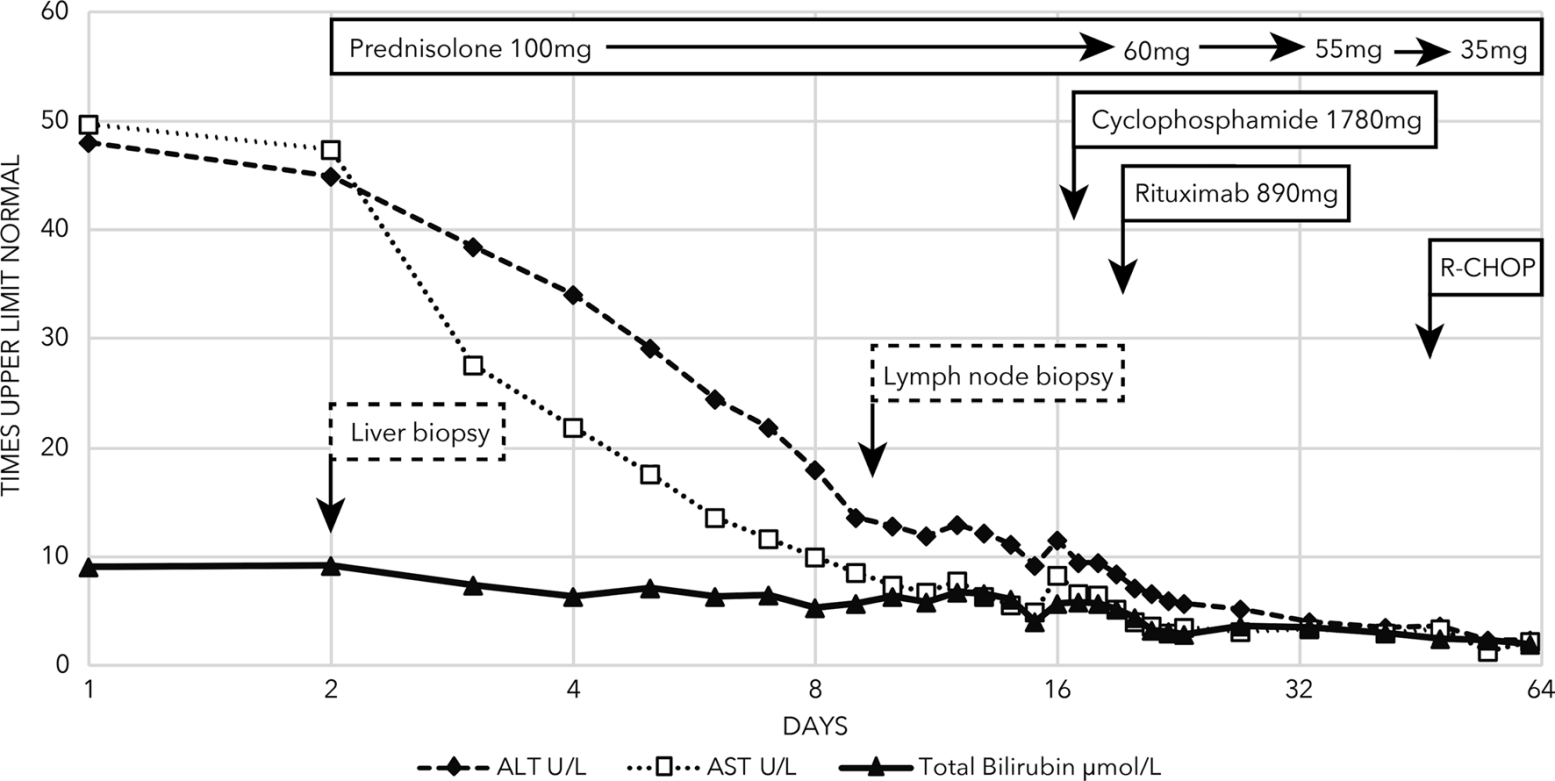
Timeline of liver function tests and treatments

Treating his lymphoma recurrence in the setting of his severe liver injury was challenging. His liver biochemistry improved slowly with oral prednisolone; by day 17 the ALT had reduced to less than 10 times the upper limit of normal and he was treated with a dose of rituximab 375 mg/m^2^ and cyclophosphamide 750 mg/m^2^ (Fig. 4). He was then discharged on a prednisone weaning plan of 5 mg per week. Four weeks later, after a treatment delay due to intercurrent COVID-19 (CV19) infection, he commenced rituximab, cyclophosphamide, doxorubicin, vincristine, and prednisone chemotherapy regimen (R-CHOP) with 75% and 50% dose reduction in doxorubicin and vincristine, respectively, due to hepatic impairment. His bilirubin normalized and ALT decreased to < 2 U/L by cycle 2 and so he continued further R-CHOP therapy at full dose. He has now been followed up for 5 months and his liver biochemistry shows a total bilirubin of 22 μmol/L, direct bilirubin 8 μmol/L, ALT 63 U/L, AST 31 U/L, ALP 144 U/L, GGT 120 U/L, and albumin 41 g/L. Interim computed tomography restaging shows complete anatomical response at all nodal sites, but with persistent splenomegaly of 18 cm.

## Discussion and conclusions

Severe liver injury is an exceedingly uncommon manifestation of Hodgkin lymphoma (HL) and is usually due to diffuse malignant infiltration. However, three distinct paraneoplastic phenomena have been described whereby HL induces severe liver injury without lymphomatous involvement of the parenchyma: vanishing bile duct syndrome (VBDS), intrahepatic cholestasis without ductopenia, referred to as HL-related idiopathic cholestasis, and acute autoimmune-mediated paraneoplastic hepatitis with fulminant hepatocyte loss. The most well described of these is VBDS due to massive cytokine release from lymphomatous cells recruiting inflammatory cells and ultimately causing interlobular bile duct destruction and characteristic portal fibrosis, with the resultant ductopenia leading to cholestasis [17, 18]. Intrahepatic canalicular stasis in the absence of bile duct destruction is thought to represent a variant of Stauffer syndrome, a paraneoplastic hepatopathy mediated partly by overexpression of interleukin-6 by the tumor cells. This was originally described in renal cell carcinoma but has since been defined in other cancers, including HL, and responds to treatment of the underlying malignancy [19–22]. The rarest is an acute paraneoplastic hepatitis with marked transaminitis, as in our case, the presumed mechanism of which is cell-mediated or humoral destruction of hepatocytes with subsequent cytokine release resulting in fulminant hepatocyte loss.

There have been two reported cases of paraneoplastic hepatitis in classical HL in the literature. Dourakis *et al*. [14] describe a case of ALF as the presenting manifestation of widespread HD that initially responded to chemotherapy, with improvement in encephalopathy and coagulopathy. The patient died from disseminated candidiasis shortly after presentation and liver histology postmortem demonstrated hepatic necrosis but no evidence of neoplastic infiltration. Similarly, Gunasekaran *et al*. [13] describe a case of a young child presenting with fulminant hepatitis and encephalopathy treated with steroids for a presumptive diagnosis of autoimmune hepatitis. He presented with recurrence of jaundice and intermittent fevers 2 months after his prednisolone was stopped and was retreated with corticosteroids as well as azathioprine. Bone marrow biopsy performed for persistent thrombocytopenia during that admission demonstrated HL. Liver wedge biopsy demonstrated confluent necrosis and an occasional microscopic focal infiltrate of HL, though the authors note that the latter would not explain the fulminant hepatic disease and that partial treatment with corticosteroids and azathioprine would be unlikely to induce HL remission.

The case we present is different to those described above in that our patient presented with NLPHL, not classical HL, and developed paraneoplastic hepatitis at the time of lymphoma recurrence rather than at diagnosis. At initial diagnosis his lymphoma was only present above the diaphragm, but when it recurred there was evidence of disease in lymph nodes draining through the portal venous system into the liver. Given this, we hypothesize that only the recurrence caused paraneoplastic hepatitis either because (a) the lymphoma cells underwent phenotypic shift generating neoantigens mimicking normal hepatocyte cell surface proteins or (b) the presentation of antigens directly to liver through the portal circulation triggered a dysregulated immune response. In the case reported by Gunasekaran *et al*. [13], disease was present below the diaphragm in the paraaortic and iliac lymph nodes, as well as in the bone marrow and the liver itself, and in the case described by Dourakis *et al*. [14], disease was present in lymph nodes above and below the diaphragm the pancreatic head, which is drained by the portal venous system. In our case, the biopsy features were not classical for a variant of autoimmune hepatitis, since plasma cells were very rare and there was prominent lymphocytic infiltration of the biliary epithelium. As such, the features were more like those seen in immune checkpoint inhibitor-induced hepatitis, raising the possibility that the HL was associated with immune dysregulation rather than a cross-reacting neoantigen.

This case highlights the difficulties posed by acute liver disease in managing patients with chemotherapy agents that have a narrow therapeutic index in this setting. Anthracyclines and vinca alkaloids both rely primarily on hepatic metabolism and require dose reduction in patients with impaired liver function [23]. In our case this was managed with “pre-phase” corticosteroids and staged introduction of rituximab and cyclophosphamide, followed by dose-attenuated doxorubicin and vincristine as the liver function improved. With this approach, the patient was able to complete the remainder of his chemotherapy at full dose with no unexpected toxicity or significant cytopenias.

In summary, we describe a case of a severe paraneoplastic hepatitis as the presenting manifestation of relapsed NLPHL that responded to treatment with oral corticosteroids. Early liver biopsy allows this phenomenon to be distinguished from diffuse malignant infiltration and facilitates treatment before life-threatening ALF develops. This is particularly salient as extrahepatic malignancy remains a relative contraindication to liver transplant and because conventional chemotherapeutic agents undergo hepatic metabolism and are contraindicated in patients with impaired liver function. As such, hepatic dysfunction must be rapidly controlled to allow the institution of targeted treatment of the underlying malignancy driving the paraneoplastic process. We propose novel mechanisms for pathophysiology of paraneoplastic hepatitis, with the presentation of antigens directly to liver through the portal circulation triggering a dysregulated immune response the most likely cause in this case.

## Data Availability

Information used for this case report can be made available from the corresponding author upon request.

## Abbreviations

ALF: Acute liver failure
ALP: Alkaline phosphatase
ALT: Alanine transaminase
AST: Aspartate transaminase
CV19: COVID-19
FDG: Fluorodeoxyglucose
GGT: Gamma-glutamyl transferase
HL: Hodgkin lymphoma
NLPHL: Nodular lymphocyte-predominant Hodgkin lymphoma
PET: Positron emission tomography
R-CHOP: Rituximab, cyclophosphamide, doxorubicin, vincristine, and prednisone chemotherapy regimen
VBDS: Vanishing bile duct syndrome

## References

[1] Kwo PY, Cohen SM, Lim JK (2017). ACG clinical guideline: evaluation of abnormal liver chemistries. Am J Gastroenterol.

[2] O'Grady JG, Alexander GJ, Hayllar KM, Williams R (1989). Early indicators of prognosis in fulminant hepatic failure. Gastroenterology.

[3] Hey P, Hanrahan TP, Sinclair M, Testro AG, Angus PW, Peterson A (2019). Epidemiology and outcomes of acute liver failure in Australia. World J Hepatol.

[4] Lee WM (2008). Etiologies of acute liver failure. Semin Liver Dis.

[5] Rowbotham D, Wendon J, Williams R (1998). Acute liver failure secondary to hepatic infiltration: a single centre experience of 18 cases. Gut.

[6] Athanasakis E, Mouloudi E, Prinianakis G, Kostaki M, Tzardi M, Georgopoulos D (2003). Metastatic liver disease and fulminant hepatic failure: presentation of a case and review of the literature. Eur J Gastroenterol Hepatol.

[7] Rich NE, Sanders C, Hughes RS, Fontana RJ, Stravitz RT, Fix O (2015). Malignant infiltration of the liver presenting as acute liver failure. Clin Gastroenterol Hepatol.

[8] Disibio G, French SW (2008). Metastatic patterns of cancers: results from a large autopsy study. Arch Path Lab.

[9] Hanamornroongruang S, Sangchay N (2013). Acute liver failure associated with diffuse liver infiltration by metastatic breast carcinoma: a case report. Oncol Lett.

[10] Karmacharya P, Bhandari N, Aryal MR, Pandit AA, Pathak R, Ghimire S, Shrestha P, Bhatt VR (2014). Before it crumbles: Fulminant hepatic failure secondary to Hodgkin's lymphoma. J Community Hosp Intern Med Perspect.

[11] Trewby PN, Portmann B, Brinkley DM, Williams R (1979). Liver disease as presenting manifestation of Hodgkin's disease. QJM.

[12] Dich NH, Goodman ZD, Klein MA (1989). Hepatic involvement in Hodgkin's disease. Clues to histologic diagnosis. Cancer.

[13] Gunasekaran TS, Hassall E, Dimmick JE, Chan KW (1992). Hodgkin's disease presenting with fulminant liver disease. J Pediatr Gastroenterol Nutr.

[14] Dourakis SP, Tzemanakis E, Deutsch M, Kafiri G, Hadziyannis SJ (1999). Fulminant hepatic failure as a presenting paraneoplastic manifestation of Hodgkin's disease. Eur J Gastroenterol Hepatol.

[15] Pelosof LC, Gerber DE (2010). Paraneoplastic syndromes: an approach to diagnosis and treatment. Mayo Clin Proc.

[16] Mieli-Vergani G, Vergani D, Czaja AJ, Manns MP, Krawitt EL, Vierling JM (2018). Autoimmune hepatitis. Nat Rev Dis Primers.

[17] Bakhit M, McCarty TR, Park S, Njei B, Cho M, Karagozian R, Liapakis A (2017). Vanishing bile duct syndrome in Hodgkin’s lymphoma: a case report and literature review. World J Gastroenterol.

[18] Hubscher SG, Lumley MA, Elias E (1993). Vanishing bile duct syndrome: a possible mechanism for intrahepatic cholestasis in Hodgkin's lymphoma. Hepatology.

[19] Massaad J, Wehbi M (2007). Cholestatic jaundice as a paraneoplastic presentation of Hodgkin's lymphoma: 447. Am J Gastroenterol.

[20] Sonbol MB, Rana V, Kenderian SS, Finnes H, Witzig TE (2014). Therapeutic options for patients with lymphoma and liver dysfunction or failure during mechlorethamine shortage. Leuk Lymphoma.

[21] Yalçın Ş, Kars A, Sökmensüer C, Atahan L (1999). Extrahepatic Hodgkin’s disease with intrahepatic cholestasis: report of two cases. Oncology.

[22] Cervantes F, Briones J, Bruguera M, Font C, Grau JM, Rozman C, Montserrat E (1996). Hodgkin's disease presenting as a cholestatic febrile illness: incidence and main characteristics in a series of 421 patients. Ann Hematol.

[23] Eklund JW, Trifilio S, Mulcahy MF (2005). Chemotherapy dosing in the setting of liver dysfunction. Oncology (Williston Park).

